# The prevalence of primary headache disorders in children and adolescents in Zambia: a schools-based study

**DOI:** 10.1186/s10194-022-01477-x

**Published:** 2022-09-09

**Authors:** Nfwama Kawatu, Somwe Wa Somwe, Ornella Ciccone, Misheck Mukanzu, Derya Uluduz, Tayyar Şaşmaz, Bengü Nehir Buğdaycı Yalçın, Christian Wöber, Timothy J. Steiner

**Affiliations:** 1 Department of Paediatrics and Child Health, School of Medicine, University of Zambia, Lusaka, Zambia; 2 University of Zambia School of Medicine, Lusaka, Zambia; 3 Neurology Department, Cerrahpaşa School of Medicine, Istanbul University, Istanbul, Turkey; 4 Public Health Department, School of Medicine, Mersin University, Mersin, Turkey; 5 Department of Neurology, Medical University of Vienna, Vienna, Austria; 6 Department of Neuromedicine and Movement Science, Norwegian University of Science and Technology, Trondheim, Norway; 7 Department of Neurology, University of Copenhagen, Copenhagen, Denmark; 8 Division of Brain Sciences, Imperial College London, London, UK

**Keywords:** Child and adolescent headache, Migraine, Tension-type headache, Medication-overuse headache, Undifferentiated headache, Epidemiology, Prevalence, Schools-based study, Sub-Saharan Africa, Zambia, Global Campaign against Headache

## Abstract

**Background:**

The Global Campaign against Headache collects data from children (6–11 years) and adolescents (12–17) to inform health and education policies and contribute to the Global Burden of Disease (GBD) study. This survey in Zambia, part of this global enquiry, was the second from sub-Saharan Africa (SSA).

**Methods:**

Following the generic protocol, this was a schools-based cross-sectional survey. We used the child and adolescent versions of the structured Headache-Attributed Restriction, Disability, Social Handicap and Impaired Participation (HARDSHIP) questionnaire, self-completed by pupils within classes, in a total of nine schools in Lusaka (urban) and Copperbelt (semi-rural). These two of Zambia’s ten provinces were selected to represent the country’s urban/rural divide. Headache diagnostic questions were based on ICHD-3 except for undifferentiated headache (UdH).

**Results:**

Of 2,759 potential participants, 2,089 (615 children [29.4%], 1,474 adolescents [70.6%]) completed questionnaires (participating proportion 75.7%). Children were therefore under-represented (mean age 13.1 ± 2.8 years), while gender distribution (1,128 [54.0%] male, 961 [46.0%] female) was close to expectation. Observed lifetime prevalence of headache was 97.5%. Gender- and age-adjusted 1-year prevalence estimates were 85.8% for all headache, 53.2% for migraine (definite 17.5%, probable 35.7%), 12.1% for tension-type headache (TTH), 14.8% for UdH, 3.3% for all headache on ≥ 15 days/month and 0.9% for probable medication-overuse headache. Headache durations were short: only 28.6% of participants with any headache, and only 10.5% of those diagnosed as probable migraine, reported usual durations of > 2 h (the threshold for definite migraine). Of the latter, 36.6% reported < 1 h, the duration criterion for UdH. There were weak associations of migraine (definite + probable) with female gender, and of TTH and headache on ≥ 15 days/month with adolescence. Headache yesterday was reported by 22.2% of the sample, 25.5% of those with headache.

**Conclusions:**

Headache disorders among young people are prevalent in Zambia. Among them, migraine is the most common, with UdH also highly prevalent. In this study there were diagnostic uncertainties, which rested to a large extent on the distinction between migraine and UdH among the many participants reporting headache of < 2 h’ duration. Similar uncertainties occurred in the first study in SSA, in Ethiopia. Because of these, we conclude only that migraine affects at least 17.5% of these age groups in Zambia, which is still a large proportion, adult prevalence in an earlier study being 22.9%. Supplementary estimates of attributed burden are needed to inform public-health and educational policies in Zambia.

## Introduction

The Global Campaign against Headache [1–4], in collaboration with the International Headache Society, is supporting a programme of schools-based studies in countries around the world to estimate the prevalence and burden of headache disorders in children and adolescents. The programme uses a standardised protocol and questionnaire [5] to collect data. This study in Zambia is the second in this programme to be published from sub-Saharan Africa, after Ethiopia [6]. No other data exist for these age groups (6–11 and 12–17 years) in Zambia, but an earlier survey among adults in this country, also supported by the Global Campaign against Headache, found high levels of headache [7].

Almost 30% of Zambia’s population are aged 6–17 [8]. Zambia overall has > 90% primary school completion level (ages 7–14), with gender parity, but there are regional variations and the level is rather lower (79%) in Lusaka, the Capital city [9]. Transition from primary to secondary school at age 14 is < 70%, with 43% net secondary school enrolment. Females are disadvantaged at this level (parity index 0.90) [9], and many adolescents, male or female, leave school at 16 years. Schools-based enquiries therefore have some inbuilt biases in this country, but remain the best way to reach these age groups [10].

As others in the programme, the study focuses on the headache disorders with public-health importance in adults (migraine, tension-type headache [TTH] and the disorders presenting as headache occurring on ≥ 15 days/month, including medication-overuse headache (MOH) [11–16]), together with undifferentiated headache (UdH). The last, with defining characteristics of mild pain of short duration (< 1 h), with or without migraine-like features [17], is understood to represent expressions of migraine or TTH by the immature brain. In studies in Turkey [17], Lithuania [18], Mongolia [19] and Ethiopia [6], UdH was prevalent in both children and adolescents.

The study has two purposes: to improve knowledge and understanding of the global burden of headache, and to inform health and educational policies in Zambia. This manuscript reports prevalences and their associations; a second will provide data on burden attributed to these headache disorders in these age groups.

## Methods

Following the generic protocol for the global programme [5], we conducted a cross-sectional survey in schools in two areas of the country, matching those surveyed in the earlier adult study [7]. Pupils completed a structured questionnaire within their school classes, under supervision.

### Ethics and approvals

Approvals of the protocol were given by the Excellence in Research and Science Converge Institutional Review Board (ERES IRB) and by the Ministries of Health and Education. Further approvals were obtained at each participating school from the school’s parent-teachers’ association.

Headteachers and class teachers at each selected school were asked, and agreed, to participate. Information sheets describing the nature and purposes of the survey, along with consent forms, were distributed to pupils in the participating schools to take home to parents or guardians, whose prior written consent for each participant was required by the terms of ethics approval. Verbal assent was also given by each participating child or adolescent.

Data were collected anonymously. Data protection legislation was complied with.

### Sampling and recruitment

The study, conducted during 2017 and 2018, was interrupted in late 2017 by a national cholera outbreak [20].

We sampled from two districts in two of the ten provinces of Zambia: the mainly urban Lusaka District of Lusaka Province and the more rural Ndola District of Copperbelt Province. We included nine schools in total to provide the numbers required in each age group, selecting these purposively (three in Lusaka, six in Ndola) to capture social diversity. Accordingly, we included both government and privately-owned schools (Table 1) and schools in areas of high- and low-density population, the latter tending to be of higher socioeconomic status. We did not survey in the purely rural areas of Ndola outskirts since most of the schools located there were girls’ boarding schools, with the majority of pupils originating from Lusaka.

**Table 1:** Selected schools by area and type

School location	School name	Ownership	Primary or secondary^a^
Lusaka District	Jacaranda Basic School	government	both
Lusaka District	Mount Makulu Secondary School	government	secondary
Lusaka District	Kabulonga Boys Secondary School	government	secondary
Ndola District	Simon Mwansa Kapwepwe School	government	both
Ndola District	Fibobe Primary School	government	primary
Ndola District	Milemu Secondary School	government	secondary
Ndola District	Ndola Trust School	private	both
Ndola District	Simba School	private	primary
Ndola District	Sopani Boys School	private	both

^a^ Primary: 7–12 years (6–12 years in private schools); secondary: 12–17 years

We surveyed entire classes in each school across the age-ranges 6–11 years and/or 12–17 years, excluding only pupils who declined to take part (or for whom parental objection was registered) or who, for any other reason, were unable to take part. We counted these as non-participants, but not pupils absent from school on the survey day since they were unavailable for inclusion.

Following published recommendations [5, 10], we aimed for a total of 2,100 evaluable participants, about 190 of each year of age 6–17, drawn approximately equally from all schools.

### Enquiry

The survey was mediated by an investigator and/or class teacher, who administered the questionnaires to pupils in their classes, giving assistance and explanation when necessary. Otherwise, pupils completed the questionnaires themselves and anonymously.

The survey instruments were the validated child and adolescent versions of the Headache-Attributed Restriction, Disability, Social Handicap and Impaired Participation (HARDSHIP) questionnaire, each with 45 questions [5]. Initial demographic enquiry and neutral headache screening questions were followed, in those reporting headache, by diagnostic questions based on ICHD-3 criteria [21] and enquiries into various components of headache-attributed burden. Enquiry timeframes were lifetime for the initial screening question and, subsequently, the preceding year, four weeks (28 days), one week (7 days) and 1 day (additional questions asking specifically about headache yesterday [HY], providing supportive data relatively free of recall error [10]).

We translated these instruments into the principal local languages, Bemba and Nyanja, following the Global Campaign’s translation protocol for lay documents [22].

In privately-owned schools, where English is taught as a written and spoken language from school grade 1 onwards, we administered the questionnaires in their original English versions. We did the same for older children in government schools, where English is similarly taught from grade 4 onwards, but for children in grades 1 to 4 we used the Nyanja version in Lusaka and the Bemba version in Ndola.

### Data management and entry

Questionnaires were removed, as they were completed, to secure storage at the Paediatric Centre of Excellence, a research centre within University of Lusaka Medical School. We performed independent double data-entry into Epi-info, then transcribed the data into Excel. Errors identified by comparison of the two entered datasets were reconciled by reference to the source data.

### Analysis

Analyses in accordance with the generic protocol [5] and following the plan of previous studies [6, 17–19] were performed at University of Mersin, Turkey.

Diagnoses were made during analysis. Following the HARDSHIP algorithm [5, 23], we first identified pupils reporting headache on ≥ 14 of the preceding 28 days. We presumed these to have headache on ≥ 15 days/month, and categorised the headache as probable MOH (pMOH) in a pupil who also reported medication use on ≥ 14 of the preceding 28 days or, otherwise, as “other headache on ≥ 15 days/month”. We then applied criteria for UdH (mild intensity and usual duration < 1 h [17]). Lastly, we applied the algorithm’s modified ICHD-3 criteria [21] for, in order, definite migraine, definite TTH, probable migraine and probable TTH [23]. In the analyses, we combined definite and probable migraine and definite and probable TTH [23].

We relied on these criteria to exclude secondary headaches (other than MOH); these were likely to feature among any remaining (unclassified) cases, and perhaps among those classified as “other headache on ≥ 15 days/month”.

In descriptive statistics we used means and standard deviations (SDs) for continuous variables and proportions (%) with 95% confidence intervals (CIs) for categorical data. We used chi-squared to evaluate differences between groups.

We calculated observed prevalence of each headache type, and adjusted these values for gender and age (in 3-year bands: 6–8, 9–11, 12–14, 15–17)) using official population statistics for Zambia [24]. To discover associations with demographic variables (gender and age group), we first used bivariate analysis, reporting odds ratios (ORs) with 95% CIs, then performed confirmatory binary logistic regression to calculate adjusted ORs (AORs).

We predicted 1-day prevalence of any headache and of each headache type from the means of responses to each of two questions: “On how many days in the last week did you have a headache?” and “On how many days in the last 4 weeks did you have a headache?” We compared these predictions with the proportions of pupils reporting HY.

In all analyses, we set significance at *p* < 0.05.

## Results

### Sample description

Of 2,759 potential participants (pupils present on the day), 2,089 completed the questionnaire (children 615 [29.4%], adolescents 1,474 [70.6%]; male 1,128 [54.0%], female 961 [46.0%]). Although sampling included 6-year-olds, they were only 0.2% of the sample (enrolment into government schools is at age 7 years).

The participating proportion was 75.7% (*N* = 2,089 questionnaires from 2,759 were completed sufficiently for analysis). It proved impossible to determine the exact population size from which we sampled (*ie*, including those not present) because class sizes varied from 25 to 65 without precise registers. Further, many questionnaires were necessarily excluded. Some were incomplete. Many young children (under 10 years) in government schools understood the local spoken language but could not read. Teachers were very helpful in reading to them, but some children followed instructions poorly, often giving more than one answer to each question. This meant, also, that questionnaire administration often took longer than the stipulated maximum of 45 min, disrupting lesson time.

With respect to locality, similar numbers of participants were from each: Lusaka District 1,018 (males 605 [59.4%], females 413 [40.6%]); Ndola District 1,071 (males 523 [48.8%], females 548 [51.2%]). While the intention was to sample more or less equally from each school, in Ndola District the required total could be achieved only by doubling the number of schools. Overall, females (50.0% expected [24], 46.0% achieved) were under-sampled, a reflection of their disadvantaged access to secondary education [9], although this happened only in Lusaka District. However, female under-sampling was exacerbated by uneven age-sampling, which itself was largely because of the invalidated questionnaires: children, who were 29.4% in the sample but make up 53.7% of the Zambian population aged 6–17 years [24], were considerably under-sampled in comparison with adolescents. Again, this happened in Lusaka District (13.9% children) rather than Ndola District (44.2% children). Overall mean age was 13.1 ± 2.8 years (range: 6–17; median 14).

### Headache

Observed lifetime prevalence (headache ever) was very high at 97.5% (95% CI: 93.3–99.9%; *n* = 2,036). Observed 1-year prevalence (any headache reported in the preceding year) was 87.3% (*n* = 1,824), slightly higher among females (89.8%) than males (85.2%; *p* < 0.01) and higher among adolescents (89.8%) than children (81.5%; *p* < 0.01) (Table 2). One-year prevalence adjusted for gender and age was 85.8% (Table 2).

**Table 2:** Crude (observed) 1-year prevalences (% [95% CIs]) of all headache and each headache type, overall and according to gender and age, and gender- and age-adjusted prevalences (*N* = 2,089)

	**All headache** (*n* = 1,824)	**Migraine** (*n* = 1,078)	**TTH** (*n* = 307)	**pMOH** (*n* = 23)	**Other headache on ≥ 15 d/m** (*n* = 58)	**UdH** (*n* = 309)
**Observed prevalences** (% [95% CI])						
Overall	87.3 [85.9–88.7]	51.6 [49.5–53.7]	14.7 [13.2–16.2]	1.1 [0.7–1.6]	2.8 [2.1–3.5]	14.8 [13.3–16.3]
Gender						
Male (*n* = 1,128)	**85.2** [83.1–87.2]	49.6 [46.7–52.5]	15.2 [13.1–17.3]	1.2 [0.6–1.8]	2.6 [1.7–3.5]	14.3 [12.2–16.3]
Female (*n* = 961)	**89.8** [87.9–91.7]	53.9 [50.8–57.1]	14.0 [11.8–16.2]	1.0 [0.4–1.6]	3.0 [1.9–4.1]	15.4 [13.1–17.7]
Age group (years)						
6–11 (*n* = 615)	**81.5** [78.4–84.6]	51.2 [47.3–55.2]	11.9 [9.3–14.5]	0.8 [0.1–1.5]	1.8 [0.8–2.9]	13.2 [10.5–15.9]
12–17 (*n* = 1,474)	**89.8** [88.3–91.4]	51.8 [49.3–54.4]	15.9 [14.0–17.8]	1.2 [0.6–1.8]	3.2 [2.3–4.1]	15.5 [13.7–17.4]
**Gender- and age-adjusted prevalences** (% [95% CI])						
Overall	85.8 [84.3–87.3]	53.2 [51.1–55.3] (definite: 17.5% [15.9–19.1]; probable: 35.7% [33.7–37.8])	12.1 [10.7–13.5]	0.9 [0.5–1.3]	2.4 [1.7–3.1]	14.8 [13.3–16.3]

*CI* Confidence interval, *TTH* Tension-type headache, *pMOH* Probable medication-overuse headache, *d/m* days/month, *UdH* Undifferentiated headache

Significant differences are emboldened (*p* < 0.01). Odds ratios and adjusted odds ratios for associations with demographic variables are in Table 4

Of headache types, migraine was the most common (observed 1-year prevalence, according to reported symptoms: 51.6% [definite 17.1%, probable 34.5%]; adjusted 53.2% [definite 17.5%, probable 35.7%]) (Table 2). Next were UdH (observed 14.8%; adjusted 14.8%) and TTH (observed 14.7% [definite 6.8%, probable 7.9%]; adjusted 12.1%) (Table 2). Headache on ≥ 15 days/month was reported by 3.9% (adjusted 3.3%), of whom 1.1% (adjusted 0.9%) also reported medication overuse (Table 2). There were 49 cases (2.3%) remaining unclassified.

We looked at the responses among those with headache driving the diagnosis of migraine (definite or probable): headache characteristics, and symptoms specific to migraine reported as usually accompanying headache (nausea, vomiting, photophobia, phonophobia) (Table 3). We also considered headache duration (more or less than 2 h). Only 28.6% of participants with any headache reported usual durations of > 2 h, the threshold for migraine in children [21]. Of those given the diagnosis of probable migraine (*n* = 721), only 76 (10.5%) reported usual durations of > 2 h, while 264 (36.6%) reported < 1 h. With the exception of unilaterality, all migraine-like characteristics were reported by well over half of participants with any headache. As for accompanying symptoms, 436 (60.5%) of those diagnosed with probable migraine reported nausea (*versus* 44.6% among all with headache), 244 (33.8%) reported vomiting (*versus* 27.4%), 445 (61.7%) reported photophobia (preference for dark) (*versus* 47.5%) and 662 (92.7%) reported phonophobia (preference for quiet) (*versus* 88.7%). None of these stood out as a principal driver.

**Table 3:** Responses among those with headache likely to drive the diagnosis of migraine (N variable between 1,801 and 1,824 because of missing responses to some questions)

**Headache characteristics**	**Response** n (%)	
	**Yes**	**No**
Duration > 2 h	522 (28.6)	1,302 (71.4)
Moderate or severe intensity	1,350 (76.0)	474 (24.0)
Unilateral	561 (30.9)	1,253 (69.1)
Throbbing	1,161 (64.5)	640 (35.5)
Aggravated by routine activity	1,085 (59.8)	728 (40.2)
Causing avoidance of routine activity	1,026 (56.6)	786 (43.4)
**Accompanying symptoms**		
Nausea	807 (44.6)	1,003 (55.4)
Vomiting	495 (27.4)	1,309 (72.6)
Photophobia	858 (47.5)	950 (52.5)
Phonophobia	1,604 (88.7)	204 (11.3)

### Demographic associations

There were no clear associations with gender, either for headache overall or for headache types (Tables 2, 4), although migraine emerged as more prevalent among females than males in binary logistic regression analysis (AOR: 1.2; *p* < 0.05; Table 4). TTH was more prevalent among adolescents than children (OR: 1.4; AOR: 1.4; *p* < 0.05), as was headache on ≥ 15 days/month (AOR: 1.8; *p* < 0.05) (Table 4). UdH prevalence fluctuated with age, with no trend in either direction, remaining between 16.2% and 17.2% of all reported headache.

**Table 4:** Associations of headache types with demographic variables (*N* = 2,089)

Variable	Migraine	Tension-type headache	All headache on ≥ 15 d/m	Undifferentiated headache
	**Bivariate analysis: Odds ratios [95% CI]**			
Gender				
Male (*n* = 1,128)	reference	reference	reference	reference
Female (*n* = 961)	1.2 [1.0–14]	0.9 [0.7–1.2]	1.1 [0.7–1.7]	1.1 [0.9–1.4]
Age group (years)				
6–11 (*n* = 615)	reference	reference	reference	reference
12–17 (*n* = 1,474)	1.0 [0.85–1.2]	**1.4** [1.06–1.9]	1.7 [0.99–3.0]	1.2 [0.9–1.6]
	**Binary logistic regression analysis: Adjusted odds ratios [95% CI]**			
Gender				
Male (*n* = 1,128)	reference	reference	reference	reference
Female (*n* = 961)	**1.2** [1.001–1.4]	0.9 [0.7–1.2]	1.15 [0.7–1.8]	1.1 [0.9–1.4]
Age group (years)				
6–11 (*n* = 615)	reference	reference	reference	reference
12–17 (*n* = 1,474)	1.0 [0.86–1.3]	**1.4** [1.05–1.85]	**1.8** [1.01–3.1]	1.2 [0.9–1.6]

*d/m* Days/month, *CI* Confidence interval, Significant values (*p* < 0.05) are emboldened

We did not attempt to compare districts because the study purposes did not require this, and the sampling disparities (age and gender) were too great.

### Headache yesterday (HY)

HY was reported by 464 pupils, 25.5% of the 1,824 with headache and 22.2% of the total sample (Table 5). Females with headache reported HY substantially more than males (31.5% *versus* 20.2%; *p* < 0.0001), but similar proportions of children and adolescents did so (27.5% *versus* 24.8%; *p* = 0.272) (Table 5). Of episodic headaches, migraine was more associated with HY than TTH (*p* = 0.001) or UdH (*p* < 0.001), in accordance with the reported headache frequencies of each type (Table 5). Headache on ≥ 15 days/month, including pMOH, was, of course, a far greater contributor proportionately to HY (59.3% [48 reporting HY out of 81 affected]).

**Table 5:** Proportions of those with headache reporting headache yesterday, overall and by age, gender and headache type, and predicted proportions, overall and by headache type (*N* = 1,817^a^)

Headache type	Headache yesterday				
	**Reported proportion** n (%)	**Predicted proportion**			
		**Mean reported headache frequency**		**Predicted headache yesterday** (%)	
		days in last week (F7)	days in last 4 weeks (F28)	calculated as F7/7	calculated as F28/28
Any headache (*N* = 1,817)	464 (25.5)	1.4 ± 1.7	2.9 ± 4.6	20.0	10.4
Male (*n* = 956)	193 (20.2)	1.3 ± 1.7	2.9 ± 4.6	18.6	8.1
Female (*n* = 861)	271 (31.5)	1.6 ± 1.7	3.0 ± 4.6	22.9	10.7
6–11 years (*n* = 501)	138 (27.5)	1.5 ± 1.9	2.6 ± 4.3	21.4	9.3
12–17 years (*n* = 1,323^a^)	326 (24.8)	1.4 ± 1.7	3.1 ± 4.7	20.0	11.1
Migraine (*n* = 1,074)	306 (28.5)	1.5 ± 1.7	2.5 ± 2.8	21.4	8.9
Tension-type headache (*n* = 306)	57 (18.6)	1.1 ± 1.4	2.1 ± 3.0	15.7	7.5
Probable medication-overuse headache (*n* = 23)	11 (47.8)	3.7 ± 2.3	20.7 ± 4.7	52.9	73.9
Other headache on ≥ 15 days/month (*n* = 58)	37 (63.8)	3.8 ± 2.1	19.3 ± 5.3	54.2	68.9
Undifferentiated headache (*n* = 308)	46 (14.9)	1.0 ± 1.6	1.2 ± 1.9	14.3	4.3

^a^ Seven adolescents (5 male, 2 female) did not respond to this enquiry

Also in Table 5 are mean headache frequencies reported in response to each of the two related questions (see Methods), and, calculated from these, the predicted rates of HY by headache type. For each of the episodic headaches, HY was reported more commonly than predicted, although the differences were large (up to threefold) only when prediction was based on the 4-week enquiry. For pMOH and other headache on ≥ 15 days/month, predictions based on the preceding week were reasonably approximated, while predictions based on the preceding 4 weeks were higher.

## Discussion

This second enquiry of its type to be reported from SSA found headache disorders to be common among children and adolescents, as did the first in Ethiopia [6] and others using similar methodology in Turkey [17], Lithuania [18], Austria [25] and Mongolia [19]. In this study, according to symptoms reported in response to the questionnaire used in all these studies [5], almost all of the young participants had experience of headache (lifetime prevalence 97.5%), and most (85.8%) had an active headache disorder (at least one episode of headache in the preceding year). These levels exceeded those found in all other studies [6, 17–19, 25]. Again according to symptoms reported, migraine was the most reported type. To this extent, this study agreed with the others (Table 6), but the estimated migraine prevalence of 53.2% (including 35.7% probable) rendered this study an outlier, raising questions as to validity. We return to this below. In other findings, UdH (14.8%) was second most prevalent, and within the range of the other studies (Table 6); TTH (12.1%) was less common than in other studies; headache on ≥ 15 days/month was evident in 3.3%, of whom 0.9% also reported overuse of medication (pMOH). Very few headaches (2.3%) remained unclassified.

**Table 6:** 1-year prevalences (% [95% CI]) of all headache, migraine, tension-type headache and undifferentiated headache in this and other studies using the same questionnaire [5]

	**All headache**	**Migraine**	**TTH**	**UdH**
Zambia (this study) (*N* = 2,089)^a^	85.8 [84.3–87.3]	53.2 [51.1–55.3]	12.1 [10.7–13.5]	14.8 (13.3–16.3)
Ethiopia [6] (*N* = 2,344)^a^	72.8 [71.0–74.6]	38.6 [36.6–40.6]	19.9 [18.3–21.5]	12.3 [11.0–13.6]
Turkey [17] (*N* = 7,068)^a^	73.7 [59.8–62.8]	26.7 [26.7–29.4]	12.9 [15.8–18.0]	29.2 [6.0–7.6]
Lithuania [18] (*N* = 2,505)^a^	76.6 [74.9–78.3]	21.4 [19.8–23.0]	25.6 [23.9–27.3]	24.0 [22.3–25.7]
Mongolia [19] (*N* = 4,266)^a^	59.4 [57.9–60.9]	27.3 [26.0–28.6]	16.1 [15.0–17.2]	6.6 [5.9–7.4]
Austria [25] (age range 10–18 years) (*N* = 3,386)^b^	75.7 [74.3–77.1]	24.2 [22.8–25.6]	21.6 [20.2–23.0]	26.1 [24.6–27.6]

*TTH* Tension-type headache, *UdH* Undifferentiated headache

^a^ Age- and gender-adjusted

^b^ Crude (observed) values

Before commenting further on these findings, we should note the study’s several limitations, since the findings need to be considered in their light. There were logistic and practical difficulties, not least the high numbers per class in some schools, and the poor literacy requiring questions to be read and explained to many children. In addition, the cholera outbreak mid-study was exceedingly disruptive [20].

On a general level, the principal limitation was uncertainty over the quality of responses. The same problem was noted in Ethiopia [6], and may to a degree be unavoidable. Mediated enquiry may mitigate this somewhat but, as we found here, far from entirely. As also noted in the Ethiopia study [6], epidemiological diagnosis of headache type in children and adolescents appears to be inherently imprecise. This is evidenced also by the many past surveys that have simply left large numbers of reported headaches undiagnosed (these are discussed in [17]). The questionnaire could not be validated in the local languages used in the study for the same reason as in the adult study [7] and in Ethiopia [6]: no headache experts in the country with availability to do this. It may be that some questions need reformulation, particularly those structured as leading questions enquiring into associated symptoms [5], although these have not proved problematic in Turkey [5, 17], Austria [5, 25], Lithuania [18] or Mongolia [19]. On the other hand, in these age groups, many headaches are evolving from an undifferentiated form [17], so diagnostic ambiguities are not unexpected. For this reason, extending the enquiry to parents or carers would carry no guarantee of greater certainty – and responses would not be better informed.

Other study limitations were on a specific level. The sampling process failed to equalise numbers across the age groups – a consequence of the practical difficulties in engaging with younger pupils described earlier. Additionally, schools-based sampling in Zambia has known inbuilt bias against female adolescents [9], for which statistical correction can be applied, but also likely (but unknown) degrees of bias against potential participants from low-income and rural areas. Zambia is not a low-income country by World Bank classification [26], but the reality on the ground in the more rural areas is of poverty. On the other hand, Zambia is relatively highly urbanised (44% against the SSA average of 41% [27]).

In the light of these limitations, we consider – and question – the high apparent prevalence of migraine (53.2%). Among the studies so far conducted within this global programme, this one is a clear outlier in this regard, surpassing even Ethiopia [6] (Table 6). Table 3 reveals that, while headache duration was ≤ 2 h in nearly three quarters, 60–76% of participants with headache reported each of three characteristics suggestive of migraine [21]. Only unilaterality, recognised as less common in young people, was reported by a minority (31%). Among accompanying symptoms, almost 90% reported phonophobia as an accompanying symptom. In Ethiopia, almost 80% did so [6]. These high proportions render this supposedly migraine-specific symptom unhelpful in differential diagnosis. Phonophobia makes no contribution to diagnosis on its own but, with 90% reporting it, the presence of photophobia (47.5%, itself a high proportion) would almost certainly mean fulfilment of one key criterion for migraine [21]. Against this, only 28.6% met the duration criterion for migraine (minimum 2 h [21]), so that most migraine diagnoses could only be probable, as two thirds were. It is, in fact, the finding of 35.7% probable migraine that is so unlikely. Over one third (36.6%) of these were reported as lasting ≤ 1 h, and the diagnosis of probable migraine rather than UdH rested entirely, therefore, on reported intensity. If these headaches were recategorized as UdH, the estimated prevalence of migraine would be reduced to 39.0%, much in line with Ethiopia’s 38.6% (Table 6), but this finding, too, was questioned as improbably high [6]. As an estimate of migraine prevalence, we can be confident only of the 17.5% definite; this estimate, however, is below all others in Table 6, and the reality is almost certainly higher.

Females reported slightly but significantly more headache overall, while differences in headache types were small (just significant for migraine [AOR: 1.2; *p* < 0.05; Table 4]). Age-dependent differences were seen only in TTH (AOR: 1.4) and all headache on ≥ 15 days/month (AOR: 1.8), both more prevalent (*p* < 0.05) in adolescents than children. UdH did not decline in prevalence with age (Table 4); neither did it decline as a proportion of all headache (children 16.2%; adolescents 17.3%). Since UdH is understood to represent expressions of migraine or TTH by the immature brain [17], an inverse relationship with differentiated headache types is expected as the latter develop with increasing age. This was seen in Turkey [17], Lithuania [18], Austria [25] and Mongolia [19], but not Ethiopia [6] – of relevance since Ethiopia is also in SSA. The explanation in both countries probably lies in the diagnostic uncertainty between migraine and UdH when headache is short-lasting and diagnosis depends, essentially, on reported intensity – subjectively, and on a rather insensitive scale of 1–3.

There was a high prevalence of headache on ≥ 15 days/month, reported by one participant in every 30 (3.3%). This was a much higher proportion than in Ethiopia (1.3% [6]), but it was given credence by the finding that 59% of these reported HY (Table 5). In the adult study in Zambia, the prevalence of headache on ≥ 15 days/month was egregiously high (11.5%), with pMOH accounting for most of it (7.1%) [7]. This was also in contrast to Ethiopia, where the adult prevalence of headache on ≥ 15 days/month was only 3.2% and of pMOH 0.7% [28]. As can be seen, pMOH accounted for most of the difference, and, in the more urbanised Zambia [27], pMOH was very largely an urban problem [7]. The messages from the present study – of public-health importance – are not only that headache on ≥ 15 days/month is already common in young people but also that its prevalence is detectably increasing with age (2.6% observed in children, 4.4% in adolescents; Table 2).

While HY was reported by well over half (59%) of those with headache on ≥ 15 days/month, this was supported by estimated 1-day prevalence based on reported mean frequency, which, in the preceding 28 days, was actually somewhat higher (70%). For the episodic headaches, however, reported headache days in the preceding one and four weeks always led to predicted proportions with HY that were lower than reported proportions (Table 5). The differences were considerably greater when predictions were based on four weeks rather than one. The same phenomenon was seen in Ethiopia [6] and Mongolia [19], where it was suggested that, while HY reporting should be free from it, recall error might increasingly influence retrospective estimates over longer recall periods. But, overall, 22.2% (464/2,089) of these young people reported HY, reflecting a presumed 1-day prevalence that is concerningly high. It is, nonetheless, a lower proportion than the 26.9% in Ethiopia [6].

Finally, we acknowledge that malaria is very common in Zambia (although much less so in Lusaka) [29]. Its symptoms conspicuously include headache, to the extent that malaria has been (and in some places still is) the default diagnosis of headache. The enquiry with respect to headaches in the preceding year established that most were episodic, recurrent and short-lasting. This would be an unlikely presentation of malaria, or of other prevalent infectious diseases of which headache is commonly symptomatic. We cannot say this of the small proportion (< 3%) reporting headache on ≥ 15 days/month, or of the 2.3% whose headaches remained unclassified.

The strengths of this study lie in the tested and validated methodology [5] and adequate sample size [10]. Limitations are identified and discussed above.

## Conclusions

Whatever the diagnostic divides, headache is very common in children and adolescents in Zambia, more so, apparently, than in adults [7]. The findings of this study in SSA add to those from Ethiopia [6], and inform educational and health policies in Zambia as well as adding to knowledge and understanding of headache in young people. Estimates of attributed burden – more important than prevalence from public-health and educational perspectives – will follow in a future manuscript.

## Data Availability

The data are held on file at University of Mersin. Once analysis and publications are completed, they will be freely available for non-commercial purposes to any person requesting access in accordance with the general policy of the Global Campaign against Headache.

## Abbreviations

AOR: Adjusted odds ratio
CI: Confidence interval
d/m: Days per month
GBD: Global Burden of Disease (Study)
HARDSHIP: Headache-Attributed Restriction, Disability, Social Handicap and Impaired Participation (questionnaire)
HY: Headache yesterday
ICHD: International Classification of Headache Disorders
LTB: *Lifting* *The* *Burden*
MOH: Medication-overuse headache
OR: Odds ratio
pMOH: Probable MOH
SD: Standard deviation
SSA: Sub-Saharan Africa
TTH: Tension-type headache
UdH: Undifferentiated headache
